# Cardiac contractility modulation increases action potential duration dispersion and decreases ventricular fibrillation threshold via β1-adrenoceptor activation in the crystalloid perfused normal rabbit heart^☆^^☆☆^

**DOI:** 10.1016/j.ijcard.2013.12.184

**Published:** 2014-03-01

**Authors:** James Winter, Kieran E. Brack, John H. Coote, G. André Ng

**Affiliations:** a Cardiology Group, Department of Cardiovascular Sciences, University of Leicester, UK; b School of Clinical and Experimental Medicine, University of Birmingham, UK; c NIHR Leicester Cardiovascular Biomedical Research Unit, Leicester, UK; d University Hospitals of Leicester NHS Trust, Leicester, UK

**Keywords:** ACh, acetylcholine, APD, action potential duration, CCM, cardiac contractility modulation, LV, left ventricle, MAPD_90_, monophasic action potential duration at 90% repolarization, NE, norepinephrine, VFT, ventricular fibrillation threshold, Cardiac contractility modulation, Non-excitatory stimulation (NES), Ventricular arrhythmia, Ventricular fibrillation, Action potential duration dispersion

## Abstract

**Background/objectives:**

Cardiac contractility modulation (CCM) is a new treatment being developed for heart failure (HF) involving application of electrical current during the absolute refractory period. We have previously shown that CCM increases ventricular force through β1-adrenoceptor activation in the whole heart, a potential pro-arrhythmic mechanism. This study aimed to investigate the effect of CCM on ventricular fibrillation susceptibility.

**Methods:**

Experiments were conducted in isolated New Zealand white rabbit hearts (2.0–2.5 kg, *n* = 25). The effects of CCM (± 20 mA, 10 ms phase duration) on the left ventricular basal and apical monophasic action potential duration (MAPD) were assessed during constant pacing (200 bpm). Ventricular fibrillation threshold (VFT) was defined as the minimum current required to induce sustained VF with rapid pacing (30 × 30 ms). Protocols were repeated during perfusion of the β1-adrenoceptor antagonist metoprolol (1.8 μM). In separate hearts, the dynamic and spatial electrophysiological effects of CCM were assessed using optical mapping with di-4-ANEPPS.

**Results:**

CCM significantly shortened MAPD close to the stimulation site (Basal: 102 ± 5 [CCM] vs. 131 ± 6 [Control] ms, P < 0.001). VFT was reduced during CCM (2.6 ± 0.6 [CCM] vs. 6.1 ± 0.8 [Control] mA, P < 0.01) and was correlated (r^2^ = 0.40, P < 0.01) with increased MAPD dispersion (26 ± 4 [CCM] vs. 5 ± 1 [Control] ms, P < 0.01) (n = 8). Optical mapping revealed greater spread of CCM induced MAPD shortening during basal vs. apical stimulation. CCM effects were abolished by metoprolol and exogenous acetylcholine. No evidence for direct electrotonic modulation of APD was found, with APD adaptation occurring secondary to adrenergic stimulation.

**Conclusions:**

CCM decreases VFT in a manner associated with increased MAPD dispersion in the crystalloid perfused normal rabbit heart.

## 1 Introduction

Cardiac contractility modulation (CCM) is an electrical device therapy being developed for the treatment of heart failure (HF), which remains a significant clinical burden despite decades of research and the development of useful drug therapy [1]. During CCM, electrical signals are applied to the ventricular myocardium to increase force and are timed to coincide with the absolute refractory period. Experimental and clinical studies demonstrate that CCM improves contractile performance, increases patient exercise capacity and improves quality of life (reviewed by Winter et al. [2]). Several studies have highlighted that CCM reverses the molecular remodelling associated with heart failure opposing the down-regulation of several calcium handling proteins (e.g. phospholamban, sarco-endoplasmic reticulum ATPase (SERCA)), increased expression of detrimental signalling factors (e.g. atrial natriuretic peptide) and fibrosis, which may contribute to its long term beneficial action [2,3].

We have previously demonstrated that the acute effects of CCM are mediated via stimulated norepinephrine (NE) release resulting in cardiac β1-adrenoceptor activation and shortening of locally recorded monophasic action potential duration (MAPD); in the crystalloid perfused normal rabbit heart [4]. The adrenergic dependence of CCM raises interesting questions regarding its neuro-cardiac action and the potential pro-arrhythmic nature of induced electrophysiological changes.

Efferent and afferent sympathetic and parasympathetic nerves richly innervate the cardiac ventricle and it has long been known that afferent nerves relay sensory information from the myocardium to the intra-cardiac and intra-thoracic ganglia, spinal cord and the brain for integration to regulate cardiac function [5]. It is recognised that local reflexes, acting through the cardiac and intra-thoracic ganglia, can modulate cardiac function on a beat-to-beat basis *without* involvement of the central nervous system [6]. It is perceivable that CCM captures both afferent and efferent nerve fibres, and that the former could engage intrathoracic ganglia or reflex activated sympathetic preganglionic neurones in the spinal cord. Furthermore, CCM may release acetylcholine (ACh) from parasympathetic nerves within the ventricle. ACh is known to antagonise NE synaptic release and antagonise post-synaptic adrenergic signalling and so it is necessary to establish whether ACh has a role in acute action of CCM (i.e. does ACh modulate the adrenergic component of CCM?).

It has long been acknowledged that adrenergic activation is pro-arrhythmic. We have previously shown that sympathetic nerve stimulation increases the susceptibility of the heart to ventricular fibrillation (VF) [7]. Given the adrenergic dependence of CCM and the pro-arrhythmic effect of positive inotropic drugs in HF, it is important to determine the effects of CCM on ventricular arrhythmia susceptibility [8].

The aims of this study were to investigate: 1) the role of pre and post-ganglionic signalling in CCM, 2) any role for ACh in modulating the acute ventricular response to CCM and 3) the effects of CCM on ventricular electrophysiology and arrhythmia susceptibility.

## 2 Methods

### 2.1 Animal welfare and ethical publishing declaration

Experiments were conducted on Adult male New Zealand White rabbit hearts (2.5–3.5 kg, *n* = 22), using the non-innervated and innervated heart preparations. All procedures were undertaken in accordance with ethical guidelines set out by the UK Animals (Scientific Procedures) Act 1986 and conformed to the Guide for the Care and Use of Laboratory Animals Published by the US National Institutes of Health (NIH Publication No. 85-23, revised 2010).

The author(s) of this manuscript have certified that they comply with the Principles of Ethical Publishing in the International Journal of Cardiology.

### 2.2 Isolation of the non-innervated heart preparation

Adult male New Zealand White rabbit hearts were isolated as previously described [4]. In brief, animals were pre-medicated with ketamine (Ketaset, 10 mg/kg, Fort Dodge, UK), medetomidine hydrochloride (Sedator, 0.2 mg/kg, Dechra, UK) and butorphanol (Torbugesic, 0.05 mg/kg, Fort Dodge, UK) (i.m.). Following stable sedation, animals were sacrificed with an overdose of pentobarbitone sodium (Sagatal, Rhone Merieux, UK; 111 mg/kg body weight, i.v.) containing Heparin (1000 IU, Multiparin, UK) delivered via the marginal ear vein. The hearts were rapidly excised, placed into ice cold tyrode solution to reduce metabolic rate and retrogradely perfused through the ascending aorta in conditions of constant flow Langendorff mode (40 ml/min) using a Gilson Minipuls 3 peristaltic pump (Anachem, UK).

### 2.3 Isolation of heart with intact autonomic innervation

**Fig. 1:**
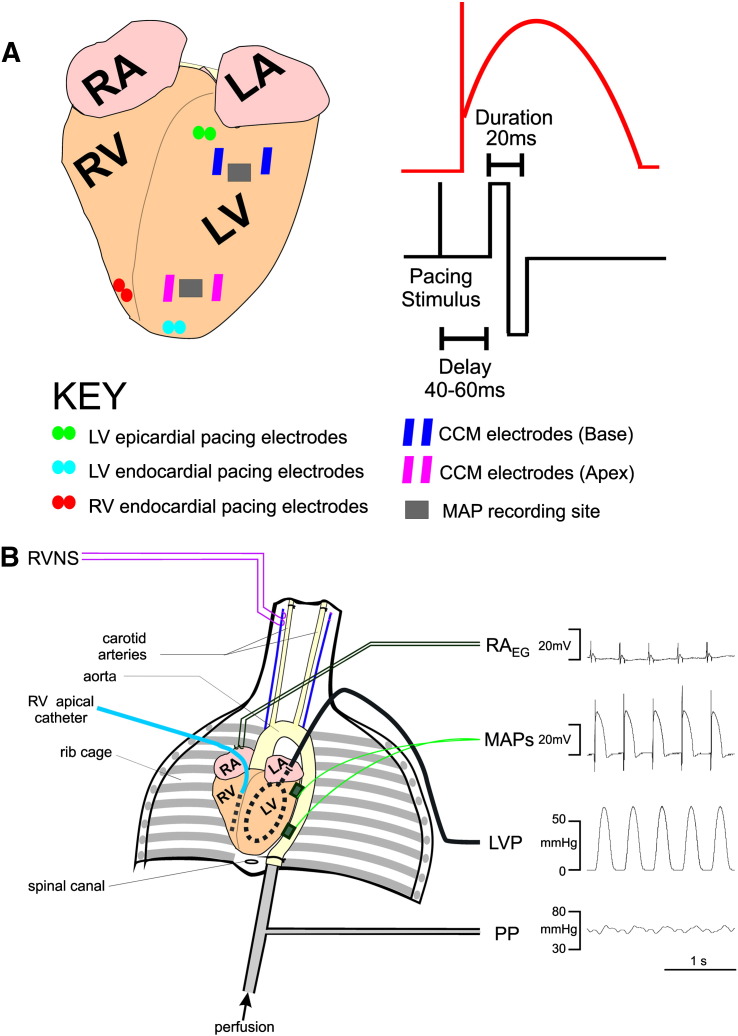
CCM signal delivery and the isolated innervated rabbit heart. A) Illustration of heart with pacing, recording and CCM stimulation sites. A square wave bipolar signal is applied directly to the surface of the left ventricle (LV) during the absolute refractory period. Signals were applied to either basal or apical regions of the LV epicardium depending upon the experimental protocol. Hearts were paced at a cycle length of 300 ms. Monophasic action potentials (MAP) were recorded in both basal and apical regions. B) Illustration of isolated innervated Langendorff rabbit heart preparation. Key: LA = left atrium, LVP = left ventricular pressure, PP = perfusion pressure, RA_EG_ = right atrial electrogram, RA = right atrium, RVNS = right vagus nerve stimulation, RV = right ventricle.

The innervated Langendorff perfused isolated heart preparation was utilised as previously described [9]. An illustration of the preparation can be found in Fig. 1B. In brief, following pre-medication, anaesthesia was maintained with i.v. propofol (5 mg as required, Rapinovet, Schering-Plough Animal Health, UK) for the remainder of surgery. Following tracheotomy animals were ventilated with room air using a small animal ventilator (Harvard Apparatus Ltd, UK; 60breaths/min). The left and right vagus nerves were isolated, and the blood vessels leading to and from the ribcage were ligated and dissected. Animals were sacrificed with an overdose of pentobarbitone sodium containing heparin (as above). The anterior portion of the ribcage was removed, and the descending aorta was cannulated. The pericardium was cut, and ice-cold Tyrode solution was applied to the surface of the heart. The preparation, extending from C1 to T12 vertebrae, was dissected from surrounding tissues and perfused through the descending aorta (100 ml/min) [9].

### 2.4 Solutions

Hearts were perfused with Tyrode solution of the following composition (mM): Na^+^ 138.0, K^+^ 4.0, Ca^2 +^ 1.8, Mg^+^ 1.0, HCO_3_^−^ 24.0, H_2_PO_4_^−^ 0.4, Cl^−^ 121.0, glucose 11.0 and acetate 20.0. The solution was continuously bubbled with 95% O_2_–5% CO_2_ to maintain a constant pH of 7.4. Temperature was maintained at 37 °C. A 3 F polypropylene catheter (Portex, UK) was inserted at the left ventricular (LV) apex for drainage of Thebesian venous effluent.

### 2.5 Functional parameters

Intra-ventricular LV pressure (LVP) was measured with a fluid-filled latex balloon connected to a pressure transducer (MTL0380, ADInstruments Ltd, UK) inserted into the LV via the left atrium. LV end diastolic pressure was maintained between 0 and 5 mm Hg. Aortic perfusion pressure (PP) was monitored with a second pressure transducer connected in series to the aortic cannula.

### 2.6 Cardiac electrical recording and pacing

MAP-TIP recording catheters (73-0150, Harvard Apparatus, UK), with a tip diameter of 1.5 mm electrode spacing of 4 mm, were used to record action potentials from the epicardial surface of the LV using a DC-coupled high input impedance differential amplifier (Joint Biomedical Workshop, University of Leicester, UK). MAPs were recorded from the LV epicardium at apical and/or basal sites depending on the experimental protocol.

Pacing electrode locations are illustrated on Fig. 1A. Endocardial pacing electrodes were inserted through the pulmonary trunk or left atrial appendage for RV and LV apical endocardial pacing, respectively (electrode diameter = 2 mm, spacing = 4 mm). For epicardial pacing, a pair of platinum hook electrodes (spacing = 4 mm) were inserted into the basal anterior LV freewall. Pacing stimuli delivered using a constant current electrical stimulator (DS7A, Digitimer Ltd, UK).

### 2.7 CCM signal generation and delivery

Square wave electrical pulses were generated using a Neurolog modular system (Digitimer, UK) with signals delivered using a constant current stimulator (Model A385, World Precision Instruments, UK). CCM stimuli were triggered using the pacing stimulus as previously described [4]. CCM signal were applied to the epicardial surface of the LV through a pair of platinum hook electrodes (inter-electrode distance ~ 1 cm) as a biphasic waveform with equal positive and negative phase amplitudes (stimulus amplitude = 20 mA and phase duration = 10 ms). We have previously demonstrated that these parameters induce optimal responses [4]. CCM signals were timed to coincide with the plateau phase using a locally recorded MAP (see Fig. 1.) and were delivered 2–3 min, allowing for a 30 second period of steady state. A 10–15 minute rest period between stimulations was used throughout the experiment for parameters to return to baseline. Hearts were paced during all CCM protocols at a 300 ms cycle length.

#### 2.7.1 Protocols

##### 2.7.1.1 Role of the intracardiac/intrathoracic ganglia

The effects of CCM on ventricular and electrophysiological performance were assessed in the *innervated Langendorff heart* in the absence and presence of the nicotinic ACh channel antagonist hexamethonium (0.5 mM) [10]. Ganglionic blockade was confirmed by inhibition of bradycardia with right vagus nerve stimulation (5Hz, 5 V). CCM was applied in basal regions of the LV.

##### 2.7.1.2 Role of ACh

The effects of CCM on ventricular and electrophysiological performance were assessed in *the non*-*innervated Langendorff heart* preparation in the absence (Control) and presence of;

A. The muscarinic ACh receptor antagonist atropine (0.1 mM) [10].
B. Exogenous ACh (1 μM) [13] with and without atropine (0.1 μM) [11].

2–3 stimulations were conducted at baseline and during perfusion. The order of perfusion was randomised from heart to heart to reduce any bias. CCM was applied in basal regions of the LV.

##### 2.7.1.3 VFT, ERP and APD dispersion

###### 2.7.1.3.1 VFT

**Fig. 2:**
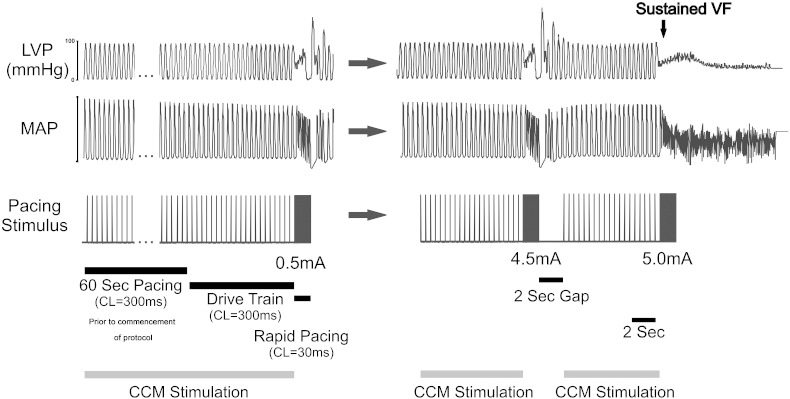
Ventricular fibrillation threshold protocol. VFT was obtained by right ventricular pacing using a train of 30 stimuli (CL = 30 ms) spanning the refractory period following a 20-beat drive train (CL = 300 ms) and determined by progressively increasing the pacing current in 0.5 mA steps, with a 2-second rest period before the next pacing train if no VF was induced. VFT was defined as the minimum current required inducing sustained VF. VFT was assessed in the absence and presence of CCM applied separately in basal and apical regions. CCM was applied for a 60-second period during constant pacing (CL = 300 ms) before commencement of the protocol, and throughout the S1 drive train. CCM was not applied during the 2-second rest period between consecutive S1 drive trains or during the rapid pacing period.

The susceptibility of the heart to VF was studied by measuring the ventricular fibrillation threshold (VFT) in the *non*-*innervated Langendorff heart*. VFT was obtained by RV endocardial, LV endocardial or LV epicardia pacing using a train of 30-stimuli (CL = 30 ms, 2 ms pulse width) spanning the refractory period following a 20-beat S1 drive train (CL = 300 ms, 2 ms pulse width) and determined by progressively increasing the pacing current (0.5 mA steps), with 2-second rest period before the next pacing train if no VF was induced (see Fig. 2). VFT was defined as the minimum current required to induce sustained VF. VFT was assessed in the absence and subsequently in the presence of CCM at basal and apical regions. CCM was applied for a minimum period of 60 s during constant pacing (CL = 300 ms) before commencing the VFT protocol. During VFT protocol, CCM was applied during the S1 beats but not during the rapid pacing period of the VF protocol. VF was terminated using a 2–3 ml bolus injection of 50 mM KCL. Hearts were allowed to beat intrinsically between protocols, allowing for a 10-minute recovery period before initiation of pacing. A 5-minute period was allowed for the stabilisation of MAPD before commencement of each protocol. In separate hearts, the effects of CCM on VFT were repeated during perfusion of the β1-adrenoceptor antagonist Metoprotol (1.8 μM) [4,9]. Baseline, Basal CCM and Apical CCM data reflects the average of 3 VFT repeats at each steady state during the experimental protocols, in each heart. The order of pacing was randomised in each experiment.

###### 2.7.1.3.2 Effective refractory period

The effective refractory period (ERP) was estimated using an extra-stimulus protocol consisting of a 20-beat drive train (S1) at a constant CL (300 ms) followed by an extra-stimulus (S2) at progressively shorter CLs (200 ms, 5 ms steps). ERP was measured in RV endocardial, LV endocardial and LV epicardial regions during basal and apical CCM. ERP was defined as the longest S1-S2 interval that failed to capture. Pacing stimuli were applied at twice the diastolic threshold current, with a pulse width of 2 ms.

###### 2.7.1.3.3 MAPD dispersion

Ventricular MAPD dispersion was measured as the difference between in MAPD the two sites using contact electrodes (*non*-*innervated hearts*). These data were obtained during the period of constant pacing (CL = 300 ms), prior to the assessment of VFT, as described above, at baseline and during CCM delivery in basal and apical regions.

##### 2.7.1.4 AP dynamics and spatial effects of CCM (optical mapping)

APs were recorded with optical mapping of the anterior LV surface of the non-innervated rabbit heart (18 × 18 mm). Optical action potentials (AP) were recorded following a bolus injection of di-4 ANEPPS (40 μl, 1 mg/ml in DMSO, Invitrogen, USA). Hearts were illuminated episcopically with an LED light source (535 nm). Emitted light was filtered through a 630 nm long-pass filter and collected using a Hamamatsu 16 × 16 element photodiode array (Cairn Research, Faversham, UK). Hearts were mechanically uncoupled with blebbistatin (5 μM). Optical studies were conducted in the non-innervated rabbit heart.

###### 2.7.1.4.1 APD dynamics

Experiments were performed to assess the dynamics to APD change during CCM. Optical APs were recorded continuously at stable baseline (300 ms CL) and during applications of biphasic CCM signals (± 20 mA, 20 ms) to the LV free wall. The effects of CCM on APD was assessed at regions within 2 mm of the stimulating electrode and in APs with a clear deflection associated with CCM stimulation.

###### 2.7.1.4.2 Spatial effects

CCM (± 20 mA, 20 ms) was applied in basal and apical regions during constant pacing (CL = 300 ms). The degree of spread of APD shortening during CCM was assessed from the site of CCM signal delivery (Base → Apex or Apex → Base). Studies were conducted in the non-innervated rabbit heart.

### 2.8 Signal measurements and analysis

Functional parameters and MAPs were recorded with a PowerLab 8 s system and digitised at 2 kHz using Chart and Scope software (ADInstruments Ltd). Optical AP signals were digitised at 2 kHz and recorded on a custom designed computer system (National Instruments, USA) using QRecord software (Dr Francis Burton, University of Glasgow, UK). LV contractile performance was assessed from an average of 20 cardiac cycles during baseline and subsequently during the steady state CCM response. Contact MAPD was measured at 90% repolarisation (MAPD_90_) averaged over 20-cardiac cycles recorded during steady state before and immediately on cessation of CCM stimulation due to signal interference from the current used during CCM. Optical APD_90_ was assessed before and during the steady state response to CCM signal delivery as there was no signal interference from the current used during CCM on the recorded APs. Both MAPD_90_ and optical APD_90_ calculations were performed using custom written analysis software (NewMap and Optiq respectively, Dr. F. Burton).

### 2.9 Statistical methods

Statistical comparisons were made using Student's paired t-tests, one- or two-way ANOVA where appropriate with Bonferroni post-hoc test. P < 0.05 was considered significant. All statistically significant differences reported in this manuscript represent a sample size to give a minimum statistical power of 0.94, at an alpha level of 0.05. Data are presented as mean values ± standard error of the mean.

## 3 Results

### 3.1 Role of intracardiac/intrathoracic ganglia

**Fig. 3:**
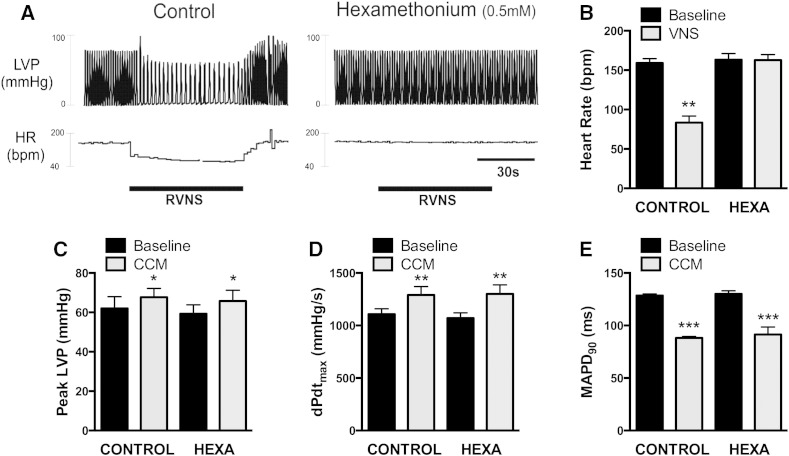
Role of ganglionic transmission in the acute ventricular effects of CCM. A) Raw data illustrating instantaneous left ventricular pressure (LVP) and heart rate (HR) during right vagus nerve stimulation (RVNS, 5Hz, 5 V) in the absence (left) and presence of 0.5 mM hexamethonium (right). B) Mean data representing the bradycardia effect of RVNS in the absence and presence of hexamethonium (n = 3). C–E) Mean data demonstrating the change in LVP (C), rate of pressure production (dPdt_max_, D) and basal MAPD_90_ (E) during basal CCM in the absence and presence of hexamethonium (n = 5). Key: BL = baseline, SS = steady state, CCM = cardiac contractility modulation. Baseline vs. stimulation (VNS or CCM); *P < 0.05, **P < 0.01, ***P < 0.001.

Data illustrating the effects of hexamethonium perfusion on the acute ventricular response to CCM, in the *innervated Langendorff heart*, are summarised in Fig. 3. Ganglionic blockade was confirmed by the abolition of right vagus nerve stimulation induced bradycardia (Fig. 3A&B). In control, right vagus nerve stimulation reduced heart rate by 76 ± 9 bpm and was abolished during perfusion with 0.5 mM hexamethonium (∆ = − 1 ± 1 bpm, n = 3). During hexamethonium perfusion, CCM stimulated enhancement of LV pressure, maximal rate of change of pressure and shortening of MAPD_90_ (Fig. 3C–E) were comparable to control (n = 5).

### 3.2 Role of ACh

#### 3.2.1 Atropine

Perfusion of atropine had no effect on the increase in LVP (∆3.9 ± 0.7 [Atropine] vs. ∆4.2 ± 0.6 [Control] mm Hg), dP/dt_max_ (∆186 ± 47 [Atropine] vs. ∆175 ± 27 [Control] mm Hg/s) or MAPD_90_ shortening (∆-27 ± 9 [Atropine] vs. ∆-28 ± 7 [Control] ms) associated with CCM (n = 5), indicating no role for endogenous ACh release in the acute ventricular effects of CCM.

#### 3.2.2 Exogenous ACh

**Fig. 4:**
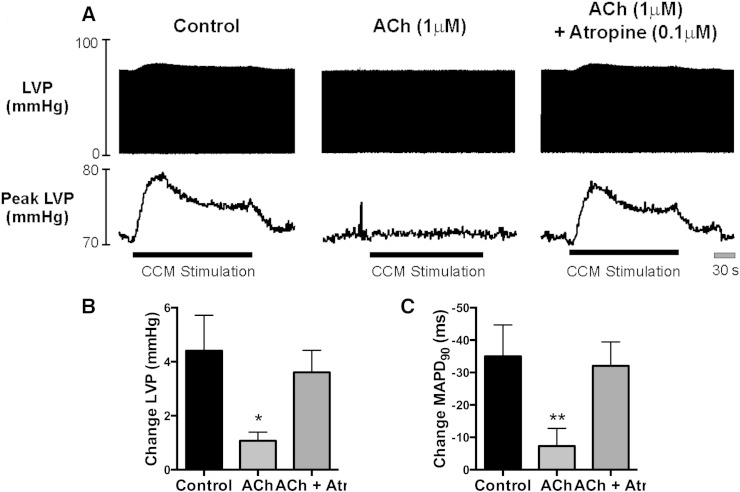
Effect of exogenous acetylcholine on the acute ventricular response to CCM. A) Raw data illustrating left ventricular pressure (LVP) and peak LVP in the absence and presence of exogenous acetylcholine (ACh, 1 μM) and ACh + atropine (0.1 μM) perfusion associated with basal CCM. B & C) Mean data demonstrating the change in peak LVP and basal monophasic action potential duration (MAPD_90_) in the absence and presence of ACh and ACh + atropine (n = 4). Difference in CCM response between experimental conditions; *P < 0.05, **P < 0.01.

Raw and mean data illustrating the effect of exogenous ACh perfusion on the acute effects of CCM are presented in Fig. 4. Exogenous ACh inhibited the increase in peak LV pressure (Fig. 4B), maximal rate of change of pressure (Δ126 ± 16 vs. Δ30 ± 4 mm Hg/s, P < 0.05) and shortening of MAPD_90_ (Fig. 4C) associated with CCM (n = 4). The inhibition of CCM with ACh was reversed during combined perfusion with 0.1 μM atropine (Fig. 4, n = 4). Exogenous ACh perfusion caused coronary vasodilation, evidenced by a reduction in PP (54.4 ± 10.9 vs. 50.9 ± 10.6 mm Hg, P < 0.05), but had no significant effect on LVP or MAPD_90_ in baseline conditions.

### 3.3 VFT, ERP and MAPD dispersion

#### 3.3.1 VFT

**Fig. 5:**
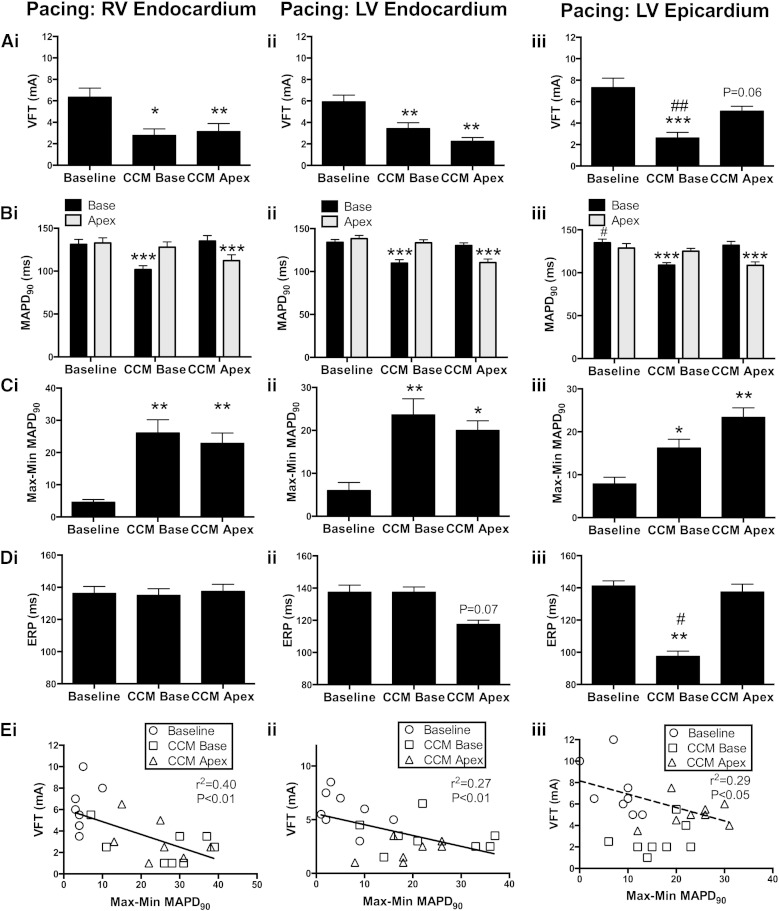
The effect of CCM on the ventricular fibrillation threshold and ventricular dispersion of monophasic action potential duration. A) Mean data showing ventricular fibrillation threshold (VFT) at baseline (BL) and during CCM at basal and apical sites during RV endocardial, LV endocardial and LV epicardial pacing. B) Mean data illustrating monophasic action potential duration (MAPD_90_) at BL and during CCM. C) Maximum–minimum MAPD_90_ at BL and with CCM. D) Effective refractory period (ERP) at baseline and during CCM E) The correlation between Max–Min MAPD_90_ vs. VFT (regression fit represents combined datasets, with the exception of (Eiii) in which basal CCM data points are excluded (dotted line)). Comparison to baseline; *P < 0.05, **P < 0.01, ***P < 0.001, base vs. apex; ^#^P < 0.05, ^##^P < 0.01 (VFT, MAPD; n = 8, ERP; n = 4, same hearts).

Mean data on the effect of CCM on VFT are illustrated in Fig. 5A. CCM was associated with a significant reduction in VFT during RV (Fig. 5Ai) and LV (Fig. 5 Aii) endocardial pacing, indicating increased susceptibility to VF. Similarly CCM applied at basal regions, during LV epicardial pacing, reduced VFT with a borderline significant trend associated with apical CCM (Fig. 5Aiii, P = 0.06). The magnitude change in VFT with apical and basal CCM was similar in all conditions, with the exception of LV epicardial stimulation where there was a larger reduction in VFT during basal vs. apical CCM (Fig. 5Aiii). Perfusion of metoprolol abolished the reduction in VFT during basal (5.35 ± 0.8 [Basal CCM + Metoprolol] vs. 5.4 ± 0.7 [Metoprolol] mA) and apical CCM (5.42 [Apical CCM + Metoprolol] vs. 5.4 ± 0.7 [Metoprolol] mA) (RV endocardial pacing, n = 6).

#### 3.3.2 ERP

Changes in ERP were dependent upon the relative location of the pacing site and site of CCM stimulation. ERP was shortened during LV epicardial pacing when CCM signals were applied to basal (close to) but not apical regions (far from) (Fig. 5Diii). Similarly there was a trend for ERP shortening, during LV endocardial pacing, with CCM applied in apical (close) but not basal regions (far) (Fig. 5Dii, P = 0.07). No effect of CCM on ERP was seen during RV endocardial pacing with either basal or apical stimulation (Fig. 5Di).

#### 3.3.3 MAPD dispersion

Mean data on the effect of CCM on MAPD_90_ and max-min dispersion are presented in Fig. 5B & C. Un-stimulated MAPD_90_ was similar between basal and apical regions during RV and LV endocardial pacing but longer in basal, vs. apical, regions during LV epicardial pacing (Fig. 5B). CCM caused significant shortening of MAPD_90_ close to the site of stimulation but not at distant sites (Fig. 5B) resulting in a significant increase of max-min MAPD_90_ (Fig. 5C) (n = 8). The increase in dispersion of repolarisation was similar during apical and basal CCM and occurred in all pacing protocols (Fig. 5C). The scatterplots presented in Fig. 5E show that VFT was inversely correlated with max-min MAPD_90_ during RV endocardial (5Ei) and LV endocardial (5Eii), but not LV epicardial (5Eiii), pacing. It is apparent that the reduction in VFT with basal CCM, during LV epicardial pacing, may be the product of increased MAPD_90_ dispersion and shortening of ERP at the site of CCM stimulation. Analysis of LV epicardial pacing, when basal CCM data are removed from the dataset, reveals a trend of correlation between VFT and max-min MAPD_90_ dispersion (Fig. 5Eiii), similar to that seen with LV and RV endocardial pacing.

Perfusion of metoprolol abolished the shortening of MAPD_90_ during basal (131 ± 4 [Metoprolol + Basal CCM] vs. 128 ± 2 [Metoprolol] ms) and apical CCM (132 ± 4 [Metprolol + Apical CCM] vs. 129 ± 5 [Metoprolol] ms). Max-min MAPD_90_ dispersion during CCM stimulation with metoprolol was similar to baseline values (4.7 ± 2.0 [Metoprotol + Basal CCM] vs. 4.2 ± 1.7 [Metoprolol] ms) (RV endocardial pacing, n = 6).

### 3.4 AP dynamics and spatial electrophysiological effects of CCM

#### 3.4.1 Optical vs. contact

Optical action potentials (n = 4) recorded at baseline under un-stimulated conditions were significantly longer than those recorded with contact electrodes (RV endocardial pacing, n = 8) in both basal (159 ± 6 [Optical] vs. 131 ± 6 [Contact] ms, P < 0.01) and apical (169 ± 6 [Optical] vs. 133 ± 6 [Contact] ms, P < 0.01) regions. Optical recordings were associated with a significant apico-basal dispersion of APD_90_ (169 ± 6 [Apex] vs. 159 ± 6 [Base] ms, P < 0.01) not present in contact electrode recordings (133 ± 6 [Apex] vs. 131 ± 6 [Base]) and attributable to the adverse electrophysiological effects of blebbistatin.

#### 3.4.2 AP Dynamics

**Fig. 6:**
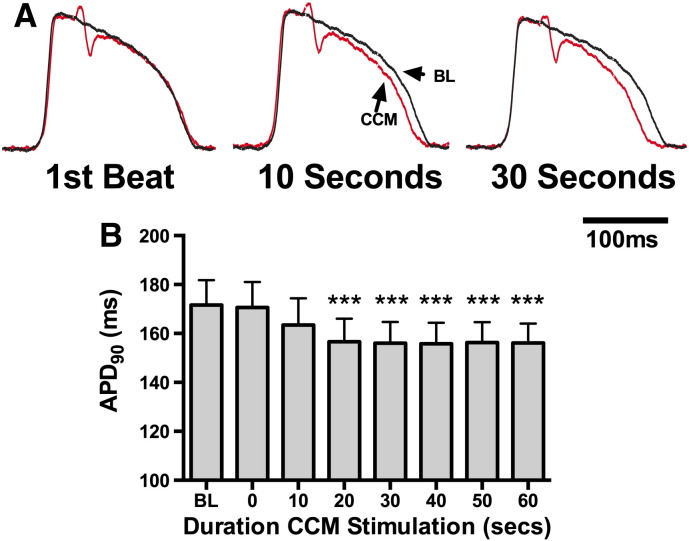
Temporal changes in action potential duration during local CCM stimulation. A) Example action potentials demonstrating the effects of CCM on action potential duration (APD_90_) on the first beat, and after 10–30 s of stimulation (optical). B) Mean data demonstrating the time course of APD_90_ changes during local CCM stimulation. CCM vs. baseline; ***P < 0.001 (n = 3).

Raw and mean data demonstrating time dependent adaptation of ventricular APD during LV free wall CCM are presented in Fig. 6. APs were recorded close to the site of CCM stimulation (within 2 mm). Despite a clear deflection attributable to the CCM stimulus (Fig. 6), there was no instantaneous effect on APD_90_. Rather, APD shortened over a period of seconds with a maximal response at 30 s. Responses recovered to baseline 2–3 min after cessation of stimulation (data not shown).

#### 3.4.3 Spatial effects of CCM

**Fig. 7:**
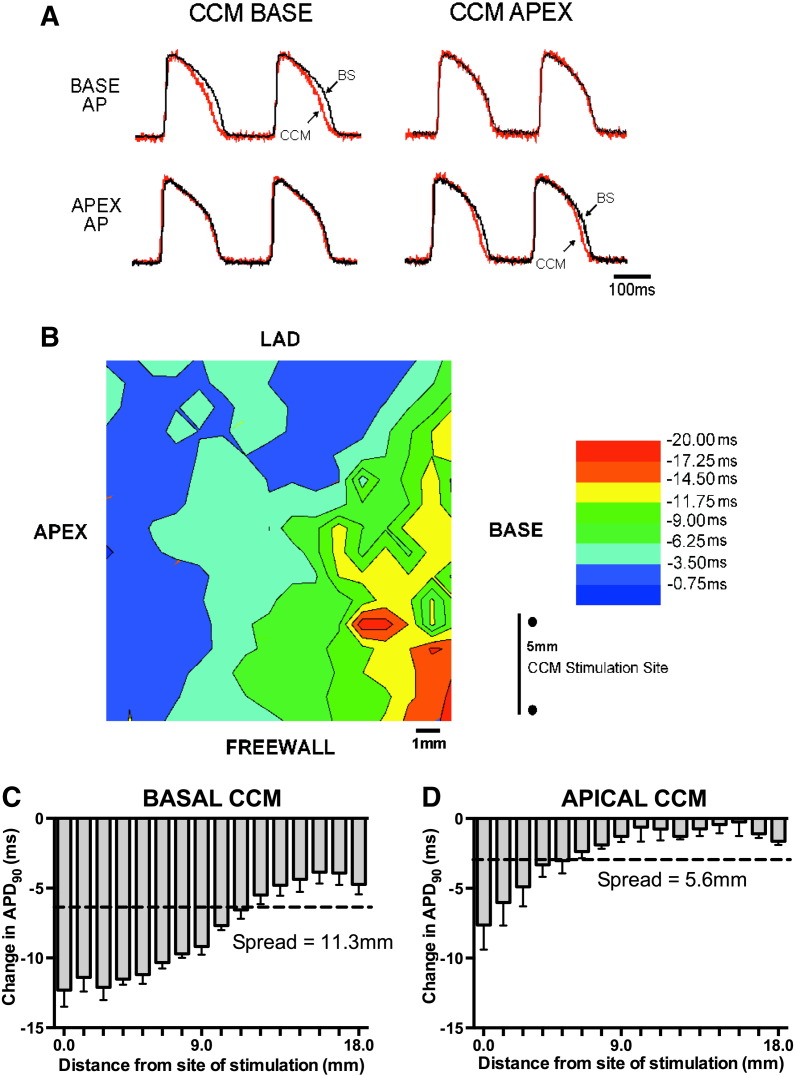
The effects of CCM on action potential duration across the anterior ventricular surface. A) Raw data demonstrating optically action potentials recorded at base and apex regions at baseline (BL) and during basal and apical CCM. B) The spatial spread of APD shortening during basal CCM from a representative experiment. D & E) Mean data demonstrating the effective range of APD shortening from the site of CCM delivery. Dotted lines denote the statistical cut off for each data set. (n = 4).

Raw and mean data demonstrating the effect of CCM on optically recorded APs are illustrated in Fig. 7A. In accordance with data obtained from contact electrodes, CCM caused APD shortening close to the stimulation site, but not at recording sites distant to CCM stimulation. Raw data illustrating the spatial spread of optical AP shortening during high amplitude basal CCM is shown in Fig. 7.and shows that the degree of APD shortening decays relatively uniformly away from the stimulation site. The range of APD shortening appears greater during basal CCM as defined by distance from site of stimulation when APD shortening fails to reach statistical significance (Fig. 7C-D, n = 4).

## 4 Discussion

To the best of our knowledge, this is the first study to demonstrate that CCM (20 mA, 20 ms) increases susceptibility to ventricular fibrillation, an effect that is dependent on β1-adrenoceptor activation and is associated with an increase in ventricular APD dispersion. In addition, we have shown that the ventricular effects of CCM are abolished during the perfusion of exogenous ACh but unaffected during muscarinic receptor antagonism and ganglionic blockade, suggesting that local intracardiac/spinal reflex pathways are not involved. These data support the notion that the acute effects of CCM are mediated through local release of NE from post-ganglionic sympathetic nerves.

### 4.1 Recruitment of intracardiac/intrathoracic ganglia

Intracardiac and intrathoracic ganglia have been shown to be involved in the beat-to-beat control of ventricular performance independently from the central nervous system [6]. The electrical current used during CCM should be sufficient to activate ventricular afferent fibres, as suggested by experiments using electrical stimulation to identify sympathetic afferents [12], and thus a proportion of the CCM response may occur through ganglionic activation and recruitment of additional efferent sympathetic inputs. We investigated this hypothesis using the ganglionic neurotransmission inhibitor hexamethonium. Although hexamethonium abolished the bradycardic response to vagal nerve stimulation it had no effect on the acute ventricular responses to CCM. These data demonstrate that the effects of CCM occur solely through the electrical stimulation of post-ganglionic sympathetic fibres that innervate the ventricle.

### 4.2 The role of acetylcholine

We confirm our previous finding that CCM increases ventricular contractility and shortens MAPD through β1-adrenoceptor activation following NE release [4]. These data are supported by studies demonstrating the adrenergic dependence of CCM in the goat and dog [13]. In the present study we investigated whether ACh, released from parasympathetic nerves with the ventricle, plays any role in the acute action of CCM.

It is well known that the vagus nerve antagonises the action of the sympathetic nervous system. Binding of NE to the β−adrenoceptors increases the activity of soluble adenylyl cyclase, catalysing the formation of cAMP, which activates protein kinase A to phosphorylate several protein targets increasing contractile force. ACh binds to muscarinic receptors reducing the release of NE from sympathetic nerves and inhibits the formation of cAMP within cardiac myocytes [14,15]. It is reasonable to assume that exogenous ACh perfusion prevents the acute actions of CCM through a combination of these mechanisms. In much the same way that CCM stimulates sympathetic nerves, parasympathetic nerves can also be stimulated. Despite substantial functional and histological evidence of parasympathetic innervation of the ventricle, atropine failed to modify the ventricular CCM response, suggesting that there was insufficient ACh release from tentative parasympathetic nerve stimulation to modulate the adrenergic component of CCM.

### 4.3 Calcium vs. hormone hypothesis

CCM was initially proposed as a technique to increase cardiac contractility through direct modulation of calcium entry during the action potential (calcium/electrotonic hypothesis). However, our data demonstrate that application of CCM signals to the ventricle stimulates the release of NE from sympathetic nerves [4], which acts upon the myocardium through classical β-adrenoceptor signalling pathways. This hormone hypothesis is supported by the results of the present study, in that both metoprolol and ACh abolished the ventricular reponses to CCM. Moreover, the positive intotropic response and shortening of MAPD during CCM are in keeping with adrenergic activation. Although we did not perform experiments with calcium channel blockers, the lack of response to CCM during both metoprolol and ACh perfusion indicates that its effects on calcium, as seen in other studies [16], are secondary to adrenergic activation.

Initial experiments describing an electrotonic modulation of action potential duration by CCM were performed in the isolated rabbit papillary muscle and utilised unipolar field stimulation. These studies demonstrate that cathodic and anodic electrical stimulation have opposing influences on force and APD, presumably due to an accumulation of electrical charge at the stimulating electrode and resultant modulation of membrane potential and L-type calcium current. However, the electrotonic hypothesis predicts that opposing effects would be exerted at the anodal and cathode electrodes and that the net change in contractility during bipolar CCM could be zero (if all regions respond comparably). Furthermore, CCM is applied in a biphasic manner and should exert equal depolarising and hyperpolarising influences at each electrode. The electrotonic hypothesis does not provide adequate explanation for the effects of CCM in whole heart and the lack of any immediate contractile effects in whole heart studies provides support for the hormone hypothesis [4,16]. Moreover, we have previously shown that CCM does not exert any effect on ventricular force during β-adrenoceptor blockade [4]. In the present study we show, using optical mapping, that biphasic CCM signals do not prolong the action potential but that APD shortens over a period of seconds, in keeping with the dynamics of adrenergic stimulation [17].

### 4.4 Regional electrophysiological response to CCM

We have previously shown that the CCM induced APD shortening was partially mediated by activation of the slow delayed rectifying potassium current (I_Ks_) [4]. Our findings are in keeping with the classical effects of sympathetic activation on ventricular electrophysiology and are supported by other studies reporting the adrenergic dependence of CCM [13]. In the present study we confirm localised APD shortening during CCM, close to the stimulation site but not in regions that are distant. The mechanisms whereby CCM exerts a greater functional effect during basal CCM and apparently “captures” a greater region is not known although it is plausible that this reflects either the pattern of sympathetic innervation, ion channel distribution and/or the effect of coronary circulation. We have previously reported a greater expression of tyrosine hydroxylase, a surrogate marker of sympathetic nerve distribution, at basal LV myocardium of the rabbit [18]. Other investigators have reported a graded distribution of catecholamines and sympathetic nerve fibres from basal to apical regions [19,20]. It is reasonable to hypothesise that CCM captures a larger number of sympathetic nerves when applied to the basal myocardium. Although we have not measured NE release in the present study we would expect that to see a greater NE release during basal CCM than with apical CCM based on the physiological parameters. We have previously shown that NE release during CCM is dependent upon the current amplitude applied to the basal LV, likely reflecting “capture” of a greater area of the LV and thus sympathetic nerves [4]. The expression of KCNQ1, the pore forming protein subunit responsible for the adrenergic sensitive slow delayed rectifier potassium current, is also higher at the base than the apex [18] which may also contribute to the greater degree of APD shortening with CCM at the base. It is also possible that the greater distance range of CCM induced shortening during basal stimulation reflects the downstream coronary circulation transporting NE away from its release site deeper and further away from the stimulation site. Because of the location, NE release in apical regions will be distributed to a smaller proportion of the myocardium via coronary circulation.

### 4.5 CCM and ventricular arrhythmias

In the present study we show that CCM signals (20 mA, 20 ms), applied to the LV epicardial surface of the normal rabbit heart, promote localised action potential shortening and give rise of non-uniformities of APD across the ventricular surface. We demonstrate that CCM increases apico-basal APD dispersion in a manner correlated with a downward shift in VFT and dependent upon b1-adrenoceptor stimulation (being abolished by metoprolol). Furthermore, ERP is substantially shortened at the site of CCM stimulation and appears to contribute to the pro-arrhythmic phenotype, causing a greater reduction in VFT when compared to the effects of MAPD_90_ dispersion alone. It is probable that the localised effects of CCM contribute to electrical instability during rapid pacing and that this underpins an increase in VF susceptibility. It is well known that spatial repolarisation heterogeneity provides an ideal substrate for the generation of ventricular arrhythmias [21]. Dispersion of APD is a known pro-arrhythmic mechanism underlying circus movement re-entry around refractory regions [22] and computational studies demonstrate a key role of APD dispersion in the genesis and propogation of VF [23].

#### 4.5.1 Clinical implications

Our data demonstrate that CCM increases the susceptibility of the crystalloid perfused rabbit heart to experimentally induced VF, however it is recognised that the present study utilises healthy non-disease animal hearts and as such we are cautious of offering direct correlations with clinical application of CCM therapy in human heart failure. Further study in animal models of heart failure, failing human tissue and prospective clinical studies would be necessary to confirm our results. It is clear that CCM signals of ± 20 mA and 20 ms applied to the rabbit heart may not be directly comparable to the application of similar or smaller amplitude signals in the larger canine or human heart and these values were selected from our previous experience and not as a comparison to clinical CCM. Nevertheless, the observed effects on action potential dispersion, regional repolarisation and VFT may be of scientific and clinical interest.

Anecdotal evidence from clinical studies of CCM suggests that there is no increase in the number of serious adverse arrhythmic events with CCM. However these trials are relatively small in size, short in duration and do not provide any record on the occurrence of arrhythmic events i.e. number of premature ventricular beats, non-sustained VT events, other than those presenting in clinic i.e. sustained VT and VF [2]. Several factors including the intermittent nature of chronic CCM therapy (4–6 h per day), the interaction of CCM with long-term beneficial remodelling, and the use of β-blockers may influence the occurrence of arrhythmias in vivo. Exercise regimes, like CCM, are associated with intermittent sympathetic activation and are widely accepted to improve patient quality of life without affecting mortality and hospitalisation rates (during a 4 year follow up period) [24]. It is attractive to suggest that CCM, which is commonly applied intermittently for 4–6 h per day, mimics the effects of daily exercise; however exercise will have many additional effects other than an increased sympathetic tone.

Whilst exercise regimes and CCM are both adrenergic in nature, it should be noted that clinical studies of CCM have been carried out in patients on β-blockers. Although the degree of β-blockade cannot be accurately assessed in each patient, there is the possibility that the level of β-blockade present could counteract at least in part the adverse effects of CCM on arrhythmogenesis. Complete β-adrenoceptor blockade is unlikely to be achieved clinically due to the association with significant side effects with potentially high doses. Rather the goal of β-blockade therapy in heart failure is to reduce sympathetic over-activation. CCM has been shown to be effective (acutely and chronically) in patients receiving β-blockade drugs [2]. It cannot be ruled out that the long-term beneficial effects of CCM are not mediated by β1-adrenoceptor signalling. It is equally plausible that the long-term beneficial effects of CCM in heart failure, for example upon molecular remodelling, offer some protection from associated arrhythmias negating the negative influence of acute CCM therapy. It is clear that more detailed study on the long-term mechanism of action of CCM is required, with a special focus on the effects of long term CCM on spontaneous ventricular arrhythmias and arrhythmias susceptibility.

### 4.6 CCM and ventricular relaxation

As with our previous study, CCM increased LV pressure development and contractility but had little effect on ventricular relaxation. This finding is in accordance with other reports [25]. We have previously proposed [4] that the localised effects of CCM and the proportion of sympathetic fibres stimulated during CCM may be insufficient to significantly alter global relaxation. Relaxation is dependent upon both calcium reuptake into the sarcoplasmic reticulum and the passive properties of the myocardium and as such may not be a sensitive to the localised effects of CCM. We cannot discount that there may be regional effects of CCM on relaxation that are not reflected in the more global measure reported in our study.

### 4.7 Ventricular fibrillation threshold

The susceptibility of the heart to VF was studied by measuring the VFT, which is a quantifiable and reproducible measure reflecting electrical stability [7]. Despite controversy regarding its ability to predict the effects of some anti-arrhythmic drugs, VFT has been shown to be a powerful predictor of altered arrhythmia vulnerability in relation to the autonomic nervous system [7,26].

### 4.8 Study limitations

#### 4.8.1 Site of CCM delivery different to clinical use and comparisons with larger species

We use epicardial LV sites for CCM delivery to ascertain the effects and mechanisms underlying CCM. Importantly, this method is in accordance with previous mechanistic studies in both the canine and ferret [33,34]. Clinically, the site of CCM delivery chosen is the RV septum [2], as it is convenient for and akin to standard cardiac device lead placement. Whilst our study differs in this regard, other investigators have reported that the effects of CCM are abolished by β-blockade during stimulation of the RV septum of the goat [13], in accord with our previous work [4]. The localised electrophysiological effects of CCM suggest that application in any region may promote heterogeneities of repolarisation and provide a substrate for arrhythmia.

Histological studies suggest that there exists a transmural dispersion of nerve distribution through the hearts, with less sympathetic innervation but more parasympathetic innervation of the ventricular endocardium [20]. We cannot discount that epicardial CCM captures more sympathetic fibres than the endocardial approach used clinically and that this will influence the change in VFT with CCM.

Although the spacing between our electrodes is proportionally similar to that used in larger species (e.g. dogs and humans) we cannot exclude that a 20 mA stimulus might stimulate a greater proportion of the smaller rabbit heart in relation to larger hearts. Despite this, the degree of inotropic enhancement is similar to that reported in other studies [16,25]. The parameters of our CCM signal were selected on the basis of our previous work characterising functional and electrophysiological responses with varying signal amplitude, delay and duration [4].

#### 4.8.2 Electromechanical uncoupling agent

During optical mapping we use the electromechanical uncoupler blebbistatin to prevent cardiac movement during imaging. We have recently reported that blebbistatin has a direct effect on ventricular electrophysiological parameters prolonging APD and increasing resistance to VF induction [27]. Due to the pronounced electrophysiological effects of blebbistatin, data on the effects of CCM on VFT were not collected in optical studies, rather these studies were used solely to provide confirmatory data on the regional influence of CCM, to complement our previously published findings [4] and add additional insight into the spatial distribution and dynamics of CCM effects. The mechanisms of action of blebbistatin's electrophysiological effects are not clear and require further investigation.

## 5 Conclusions

To our knowledge, this study represents the first direct investigation of the effects of CCM on ventricular arrhythmia vulnerability. Our data show that CCM (± 20 mA, 20 ms) increases the vulnerability to ventricular fibrillation through a β1-adrenoceptor dependent mechanism and is associated with APD shortening and an increase in APD dispersion (in the crystalloid perfused rabbit heart). In conjunction with our previous study, we conclude that the acute effects of CCM are mediated via NE release from efferent postganglionic sympathetic nerves as the response to CCM can be abolished by exogenous ACh perfusion.

## Disclosures

None.
